# Glycaemic abnormalities induced by small molecule tryosine kinase inhibitors: a review

**DOI:** 10.3389/fphar.2024.1355171

**Published:** 2024-02-01

**Authors:** Takudzwa Mugiya, Mamosheledi Mothibe, Andile Khathi, Phikelelani Ngubane, Ntethelelo Sibiya

**Affiliations:** ^1^ Pharmacology Division, Faculty of Pharmacy, Rhodes University, Makhanda, South Africa; ^2^ School of Laboratory Medicine and Medical Sciences, University of KwaZulu-Natal, Durban, South Africa

**Keywords:** diabetes mellitus, small molecule tyrosine kinase inhibitors, insulin resistance, blood glucose, cancer

## Abstract

In light of the expected increase in the prevalence of diabetes mellitus due to an aging population, sedentary lifestyles, an increase in obesity, and unhealthy diets, there is a need to identify potential pharmacological agents that can heighten the risk of developing diabetes. Similarly, it is equally important to also identify those agents that show blood glucose-lowering properties. Amongst these agents are tyrosine kinase inhibitors used to treat certain types of cancers. Over the last two decades, there has been an increase in the use of targeted chemotherapy for cancers such as renal cell carcinoma, chronic leukaemia, and gastrointestinal stromal tumours. Small molecule tyrosine kinase inhibitors have been at the forefront of targeted chemotherapy. Studies have shown that small molecule tyrosine kinase inhibitors can alter glycaemic control and glucose metabolism, with some demonstrating hypoglycaemic activities whilst others showing hyperglycaemic properties. The mechanism by which small molecule tyrosine kinase inhibitors cause glycaemic dysregulation is not well understood, therefore, the clinical significance of these chemotherapeutic agents on glucose handling is also poorly documented. In this review, the effort is directed at mapping mechanistic insights into the effect of various small molecule tyrosine kinase inhibitors on glycaemic dysregulation envisaged to provide a deeper understanding of these chemotherapeutic agents on glucose metabolism. Small molecule tyrosine kinase inhibitors may elicit these observed glycaemic effects through preservation of β-cell function, improving insulin sensitivity and insulin secretion. These compounds bind to a spectrum of receptors and proteins implicated in glucose regulation for example, non-receptor tyrosine kinase SRC and ABL. Then receptor tyrosine kinase EGFR, PDGFR, and FGFR.

## 1 Introduction

Diabetes mellitus (DM) is a chronic metabolic disorder characterised by elevated blood glucose concentration due to insulin resistance, deficiency of insulin, or both (Bastaki, 2005). DM affects the metabolism of fats, carbohydrates, and proteins, resulting in chronic hyperglycaemia (Bastaki, 2005; Saeedi et al., 2019). The global prevalence of DM is on the rise (Bastaki, 2005; Saeedi et al., 2019). The increase in DM prevalence may be attributed to sedentary lifestyles and diets rich in refined carbohydrates (Bastaki, 2005; Saeedi et al., 2019; Sibiya et al., 2022). Diabetes can be classified into type 1, type 2, and gestational diabetes mellitus (Bastaki, 2005; Saeedi et al., 2019; Sibiya et al., 2022). Reports have estimated that 90% of all DM cases are type 2 (Saeedi et al., 2019; Sibiya et al., 2022). An estimated 171 million people were affected by DM before 2010 and this figure is predicted to increase to 300 million by 2025 (Sibiya et al., 2022). An estimated 5.2 million deaths globally per year have been attributed to DM, exerting pressure on health systems worldwide (Glovaci et al., 2019). Many routinely prescribed pharmacological agents are believed to induce glucose intolerance and/or cause DM in non-diabetic individuals or they can negatively impact glycaemic control in diabetic patients (Bressler and DeFronzo, 1994). These effects on glycaemic control by pharmacological agents can be attributed to the induction of insulin resistance, inhibition of insulin secretion, and direct cytotoxic effects on pancreatic cells (Fathallah et al., 2015). Patients with diabetes often have other coexisting medical conditions which often leads to polypharmacy (Alwhaibi et al., 2018). The use of polypharmacy has the potential to worsen glucose tolerance (Dobrică et al., 2019). Several pharmacological agents influence glucose metabolism, including certain antihypertensives, antiretrovirals, atypical antipsychotics, antibiotics, and immunosuppressives (Izzedine et al., 2005). Literature indicates that patients being treated with β-blockers, protease inhibitors, thiazide diuretics, and atypical antipsychotic drugs are at a higher risk of developing DM as the prevalence of hyperglycaemia is higher with these agents (Izzedine et al., 2005). Due to the heightened risks of developing DM to date, there is a need for more antidiabetic options to be available for physicians to utilize in the management of diabetes. Interestingly, anticancer agents known as small molecule kinase inhibitors have shown mixed observations, where some generations have been reported to be associated with blood glucose-lowering effects in patients with cancer, whilst others have been associated with hypoglycaemia. This, therefore, highlights the necessity for more research regarding these agents and their effect on glucose metabolism, thus allowing a deeper insight in terms of their mechanisms of action at both cellular and molecular levels. Where consensus and a solid understanding of these agents on glucose handling is achieved, clear insights on their role in worsening or managing hyperglycaemia can be realised. Considering the burden exerted by DM, together with the time and challenges it takes to discover and develop new antidiabetics, there is merit for studies looking into leveraging on the existing hypoglycaemic effects demonstrated by small molecule tyrosine kinase inhibitors. With a defined mechanism of action, and structure activity relationship, small molecule tyrosine kinase inhibitors can potentially be repurposed for diabetes management, a route that could be time and cost-effective. Small molecule tyrosine kinase inhibitors are already in use and registered for the use of the treatment of cancers; therefore, safety and release profile studies are already available for these drugs. For a better understanding of small molecule tyrosine kinase inhibitors on glucose metabolism, firstly we will start by briefly outlining key processes involved in glucose homeostasis.

## 2 Glucose metabolism

### 2.1 Insulin secretion

Insulin secretion is primarily glucose-induced, with other nutrients such as amino acids and fatty acids augmenting the glucose-induced secretion (Rorsman and Braun, 2013). Glucose-induced insulin secretion requires the intracellular uptake of glucose and its metabolic oxidation. The primary transporters of glucose in βcells are glucose transporter 1 (GLUT1) and GLUT3 (McCulloch et al., 2011). Insulin is produced in the rough endoplasmic reticulum as prepro-insulin, a 110 amino acid precursor. It is then packed in the Golgi apparatus as proinsulin after the removal of 24 residue signal sequence from prepro-insulin. Insulin is stored in immature secretory granules (Goodge and Hutton, 2000). Studies of isolated islets with radioactive leucine showed that granules synthesised last are the most likely to be released when β-cells are stimulated (Gold et al., 1982). The breakdown of insulin is by crinophagia and the intracellular destruction of insulin is by lysosomes (Schnell et al., 1988). The secretory granules containing insulin also contain approximately 50 polypeptides and low molecular weight compounds like GABA, glutamate, and serotonin. The granules also contain high concentrations of metal ions like Zn^2+^ and Ca^2+^ (Ashcroft and Rorsman, 1989). The secretion of insulin is biphasic, with glucose providing a triggering action and generating triggering and amplifying signals in β-cells (Henquin, 2000). β-cells are electrically excitable and utilize changes in their membrane potential to link changes in plasma glucose concentrations with the stimulation or inhibition of insulin secretion (Rorsman and Braun, 2013). β-cells have ATPsensitive K^+^ (K_ATP_) channels which at low glucose concentrations maintain a negative membrane potential and close in response to glucose stimulation (Gromada et al., 1998). Insulin secretion also involves ATP production, glucose metabolism, Ca^2+^-dependent action potential firing, and membrane depolarization (Eliasson et al., 2008). β-cell electrical activity leads to elevation of Ca^2+^ level which triggers exocytosis of insulin granules. This is referred to as Ca^2+^-dependent exocytosis of insulin granules (Henquin et al., 2006). Plasma glucose concentration homeostasis is under the feedback loop control of insulin through alterations in K_ATP_ channel closure, β-cell metabolism, and electrical activity (Eliasson et al., 2008).

Pharmacological agents that act by depolarization and Ca^2+^ influx into β-cells lead to desensitization of insulin secretion, which is a reversible state of decreased insulin secretion responsiveness of β-cells due to prolonged exposure to stimuli. Other stimuli include glucose and free fatty acids (Rustenbeck, 2002). Sulphonylureas, namely, glibenclamide and tolbutamide, cause recoverable impairment of insulin secretion (FILIPPONI et al., 1983). Studies have shown that prolonged sulphonylurea exposure of isolated islets reduced glucose-induced insulin secretion by approximately 50% (Anello et al., 1999). Calcineurin inhibitors including tacrolimus and cyclosporine are implicated as diabetogenic drugs. Cyclosporine is believed to alter mitochondrial permeability transition pore function (Chakkera et al., 2017).

### 2.2 Insulin signaling

**FIGURE 1:**
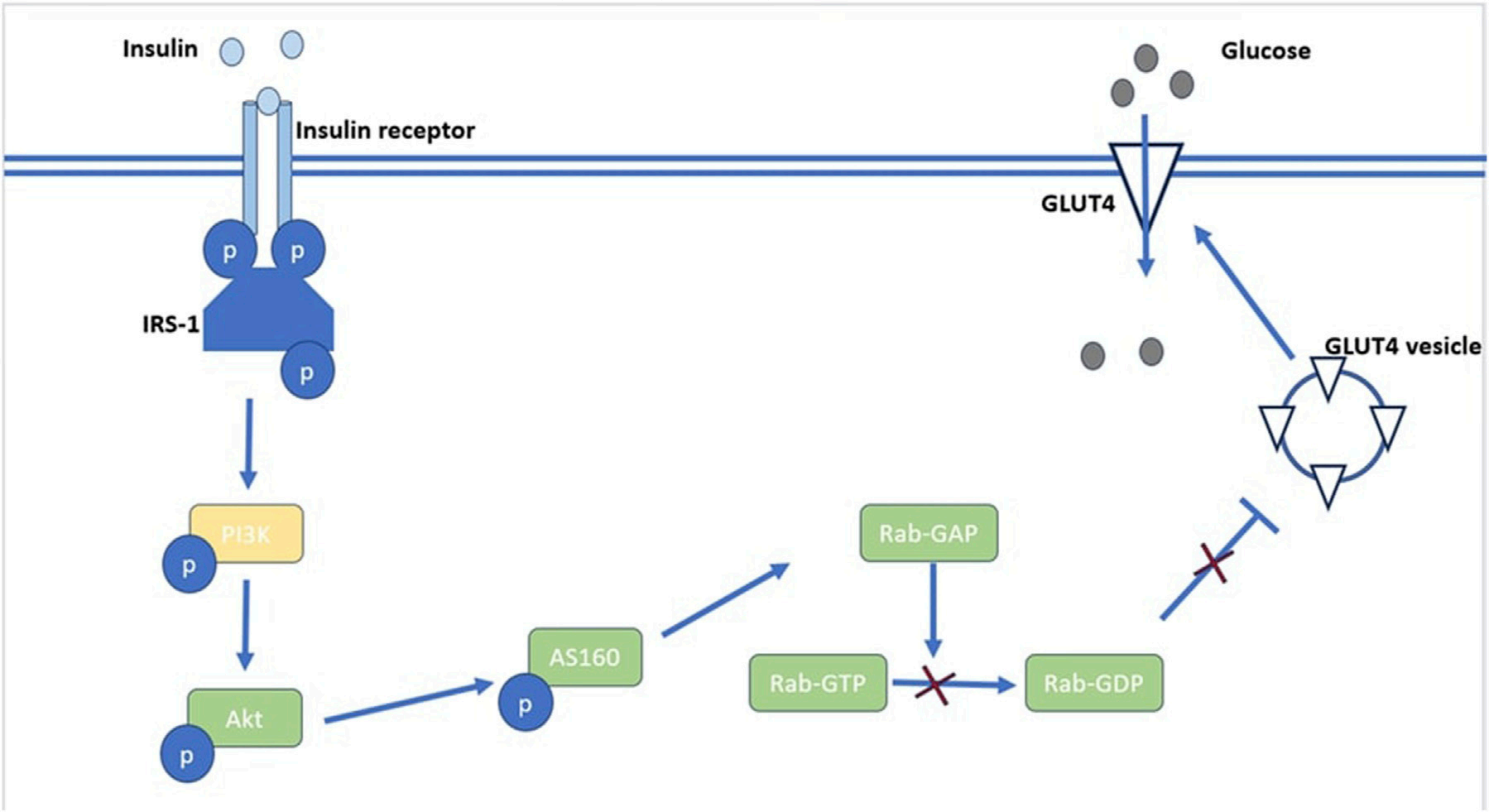
An illustration of Akt’s role in the insulin signaling pathway. Akt activation leads to phosphorylation of AS160 which reduces Rab-GAP activity resulting in reduced production of RabGDP which inhibits the translocation of GLUT4 vesicles.

Insulin receptors are present on every cell, but they are primarily expressed in skeletal muscle, liver, and fat tissue, which are the predominant insulin target sites. Insulin binds to target cell surface receptors; the insulin receptor is a heterotetramer consisting of β subunits and α subunits linked together through disulphide bonds (Bhattacharya et al., 2007). As illustrated in Figure 1, Insulin binds to cell surface protein receptors, resulting in tyrosine phosphorylation and subsequent phosphorylation of the insulin receptor substrate. This recruits and activates PI3 kinase and its downstream target Akt/PKB (Mora et al., 2004). The activation of Akt/PKB leads to phosphorylation of AS160, resulting in a reduction in Rab-GAP activity. This results in the translocation of GLUT4 and glucose uptake as depicted in Figure 1 (Sakamoto and Holman, 2008). The serine/threonine kinase Akt signaling plays a major role in insulin stimulated glucose uptake in adipose and muscle tissue. Akt signaling also inhibits glucose release from hepatocytes (Mackenzie and Elliott, 2014). The uptake of glucose via Akt signaling occurs through translocation of GLUTs to the cell membrane upon activation of insulin receptors in the presence of glucose (Jiang et al., 2003). Therefore, disruptions in PI3K/Akt/AS160 transduction pathways results in the reduction of glucose uptake in skeletal muscle. Protein tyrosine phosphatases (PTPs) can be classified into several sub-families based on their functional and structural characteristics. The three major sub-families include receptor-type PTPs (RPTPs), non-receptor-type PTPs (NRPTPs), and dual-specificity phosphatases (DSPs) (Li et al., 2016). LAR is a member of the RPTPs family and PTP-1B, PTP-1C, and PTP-1D belong to the NRPTPs family (Tao et al., 2001). From the PTPs mentioned above, only PTP-1B is a target for obesity and diabetes treatment as PTP dephosphorylation activates both leptin and insulin signaling pathways (Goldstein, 2001). The negative regulatory effect of PTP-1B on the insulin signaling pathways is a therapeutic potential in type 2 DM (Zhao et al., 2018). Patients with type 2 DM have disrupted GLUT4 expression and this contributes to insulin resistance (Albrecht et al., 1993). Disrupted intracellular signaling of GLUT4 translocation from intracellular vesicles to the cell membrane is responsible for resistance to insulin-stimulated glucose transport (Bryant et al., 2002). Apart from insulin mediated glucose transport, basal glucose uptake in most cells is facilitated by the presence of GLUT 1 (Medina and Owen, 2002).

## 3 Tyrosine kinases and cancer

**FIGURE 2:**
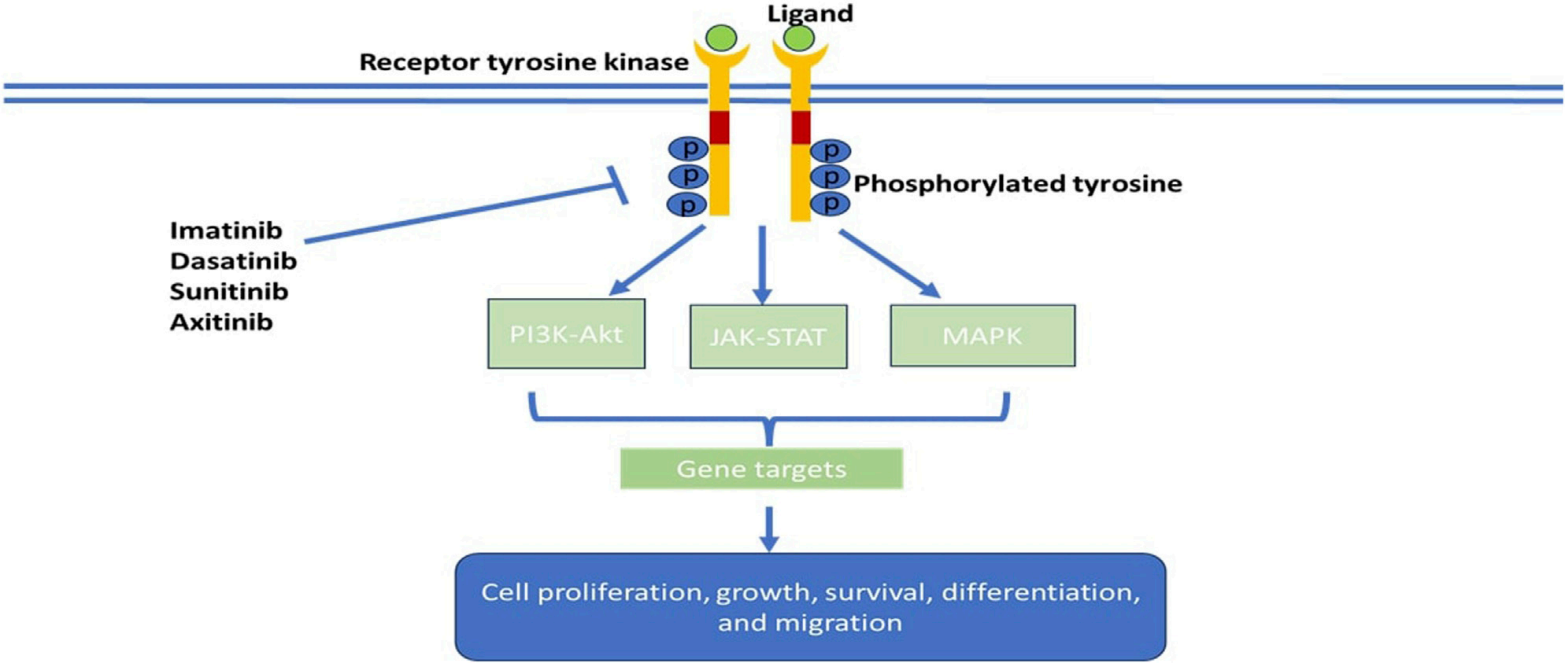
Schematic model of tumorigenic signaling pathways and their inhibition by anti-cancer- tyrosine kinase inhibitors.

Tyrosine kinases are enzymes that selectively phosphorylate tyrosine residues in specific proteins by transferring a phosphate group from ATP to the tyrosine residue. They alter the signaling cascade which modifies cell growth, differentiation, migration, death, and apoptosis, as illustrated in Figure 2 (Thomson et al., 2022). Tyrosine kinase signaling pathways are typically responsible for preventing dysregulated cell proliferation and play a role in sensitising cells to apoptotic stimuli. In cancer cells, tyrosine kinase signaling pathways are often epigenetically or genetically altered, which gives cancer cells a selection advantage (Paul and Mukhopadhyay, 2004). Tyrosine kinases comprise the major group of all oncoproteins (Rodrigues and Park, 1994). Out of the 518 kinases found in the human kinome, approximately 90 are tyrosine kinases (Bardelli et al., 2003). Therapeutic agents that are utilized in diseases linked to unusual activation of tyrosine kinase resulting in abnormal downstream oncogenic signaling are at the forefront as targets for chemotherapy (Baselga, 2006). Tyrosine kinases are primarily classified as receptor tyrosine kinases (RTKs), e.g., fibroblast growth factor receptors (FGFRs), epidermal growth factor receptors (EGFRs), platelet-derived growth factor receptors (PDGFRs), and insulin receptors (IRs) and non-receptor tyrosine kinases (NRTKs), e.g., Janus kinases (JAKs) and proto-oncogene tyrosine-protein kinase SRC (Hunter, 1995). Receptor tyrosine kinases are more than cell surface transmembrane receptors, as they are also enzymes that have intracellular kinase activity (Hunter, 1995). Non-receptor tyrosine kinases are cytoplasmic proteins varying in structure (Schenk and Snaar-Jagalska, 1999).

### 3.1 Tyrosine kinase mechanism of action

Similar to an insulin receptor activation, ligands bind to the receptor tyrosine kinase resulting in its conformational change; which enables the autophosphorylation of tyrosine residues (Figure 1). Activated receptors attract various signaling molecules which leads to the activation of kinases like the Serine/threonine kinase which leads to the phosphorylation and activation of mitogen-activated protein kinase thereby activating extracellular signal-regulated kinases 1 and 2 (Banning et al., 2014). Ligands such as epidermal growth factor (EGF) and platelet-derived growth factor (PDGF) are extracellular signaling molecules that induce receptor dimerization (Paul and Mukhopadhyay, 2004). Two types of ligand-receptor complexes can be formed, viz., one ligand may bind to two receptors resulting in a 1:2 ligand: receptor complex or two ligands can simultaneously bind to two receptor molecules resulting in a 2:2 receptor complex, for example, EGF: EGFR (epidermal growth factor: epidermal growth factor receptor) and VEGF: VEGFR (vascular endothelial growth factor: vascular endothelial growth factor receptor) (Paul and Mukhopadhyay, 2004; Hubbard and Miller, 2007). The activation loop has an open conformation after tyrosine phosphorylation and this gives access to ATP and substrates; ATP from Mg-ATP is transferred to the tyrosine residue (Schlessinger, 2000). The ATP binding sites function as a docking site for numerous downstream signaling proteins containing SRC homology-2 (SH2) or protein tyrosine binding (PTB) domain (Du and Lovly, 2018). These docking proteins recruit other effector molecules which contain SH2, SH3, PTB, and Pleckstrin homology domain (Paul and Mukhopadhyay, 2004; Du and Lovly, 2018). As a result, signaling complexes are formed to an activated receptor and membrane which leads to activating a cascade of intracellular biochemical signals resulting in activation or regression of genes and this is what defines biological responses to signals (Östman and Böhmer, 2001). Receptor tyrosine kinases transfer complex information relating to cell growth from the cell’s external environment to the cell nucleus thus activating transcriptional pathways which control cellular functions (Du and Lovly, 2018).

### 3.2 Tyrosine kinase inhibitors

Cancer growth is usually promoted by the activation of mutations or gene amplifications of specific serine kinase or tyrosine kinases (Cheng and Force, 2010). The non-specificity of traditional chemotherapy drugs, impacting both cancerous and healthy cells, lead to a range of side effects and pose a narrow therapeutic index and potential drug resistance. Tyrosine kinase inhibitors in cancer therapy offer the advantage of selectively targeting and disrupting the upregulated activities of enzymes involved in cancer cell signaling, providing a more precise and less toxic treatment approach (Arora and Scholar, 2005). The first tyrosine kinase inhibitor approved by the FDA was imatinib in 2001. By 2015, 28 small molecule tyrosine kinase inhibitors had been approved by the FDA, and by 2021 the FDA had approved 73 small molecule kinase inhibitors (Wu et al., 2015; Ayala-Aguilera et al., 2022). Tyrosine kinase inhibitors have two classes, small molecules that target specific kinases and monoclonal antibodies that target receptor kinases or their ligands (Cheng and Force, 2010). Tyrosine kinases play an important role in modulating growth factor signaling (Arora and Scholar, 2005). Tyrosine kinase inhibitors are orally active and work by competitive ATP inhibition at catalytic binding sites of oncogenic tyrosine kinases, as illustrated in Figure 2. Tyrosine kinase inhibitors differ from each other through targeting different kinases, differing pharmacokinetics, and adverse effects. Skin toxicity is an adverse effect observed in more than 50% of the patients on tyrosine kinase inhibitors (Hartmann et al., 2009). The use of tyrosine kinase inhibitors is limited by the development of resistance and lack of tumor response in the general population (Arora and Scholar, 2005).

### 3.3 Tyrosine kinase inhibitors mechanism

Tyrosine kinase inhibitors are categorized by their binding modes into two classes: irreversible and reversible. Irreversible kinase inhibitors form covalent bonds with reactive nucleophilic cysteine residue proximal to the ATP binding site, blocking the ATP site resulting in irreversible inhibition (Wu et al., 2015). Reversible kinase inhibitors can be categorised into five types based on the conformation of the binding pockets and the Asp-Phe-Gly DFG motif which is a conserved amino acid sequence from the activation loop (Noble et al., 2004). Type I inhibitors competitively bind to ATP binding sites on active kinases that have aspartate residue of the DFG motif facing the catalytic site of the kinase. Type I½ inhibitors bind to a DFG-Asp in inactive conformation (Roskoski, 2016). Type II inhibitors bind to inactive kinase with the aspartate residue protruding outward from the ATP binding site (Noble et al., 2004; Wu et al., 2015). Type I, I½, and II inhibitors reside in a part of the adenine binding pocket (Roskoski, 2016). Type III inhibitors only bind to allosteric pockets next to the ATP binding site as they do not interact with or alter the ATP binding pocket (Noble et al., 2004). Type IV inhibitors bind to allosteric sites that are not on the ATP binding pocket (Cox et al., 2010). Type V includes kinase Inhibitors like bisubstrate and bivalent inhibitors which display multiple binding modes (Cox et al., 2011). Irreversible kinase inhibitors are known as type VI inhibitors. Tyrosine inhibitors can inhibit either receptor or cytosolic tyrosine kinases. They compete with ATP for binding sites on tyrosine kinase (e.g., imatinib, gefitinib, PP1-AG1872) (Table 1). Other tyrosine kinase inhibitors such as lavendustin A exert their effect through allosteric inhibition on tyrosine kinase. Some tyrosine kinase inhibitors inhibit tyrosine kinase interaction with other proteins, including UCS15A. Tyrosine kinase inhibitors such as cetuximab inhibit ligands from binding to receptor tyrosine kinases (Cismowski et al., 2007).

**TABLE 1:** Summary of small molecule tyrosine kinase inhibitors investigated for their antidiabetic activitiy.

Small molecule tyrosine kinase inhibitors	Mechanism of action	Pharmacological uses	Glycaemic effect
Imatinib	Binds to ATP binding site of the ABL domain of the BCR-ABL (Roskoski, 2003)	Treatment of Philadelphia chromosome positive (Ph^+^) chronic myeloid leukaemia and gastrointestinal stromal tumours (Druker et al., 2001)	Hypoglycaemic (Breccia et al., 2004)
Axitinib	Selectively inhibits VEGFR-1, -2, and -3, and0 also inhibits PDGFRβ and KIT. Its benzamide group forms H-bonds with αC-Glu885 and the DFG-Asp1046 on the juxta-membrane of the VEGFR (McTigue et al., 2012)	mmjn Treats advanced renal cell carcinoma (Ayala-Aguilera et al., 2022)	Hypoglycaemic (Tyrrell and Pwint, 2014)
Nilotinib	Selectively inhibits autophosphorylation of BCR-ABL (Weisberg et al., 2005)	Treatment against imatinib-resistant mutants of BCR-ABL (Weisberg et al., 2006)	Hyperglycaemic (Racil et al., 2013)
Gefitinib	Inhibit the activation of ErbB-1 (EGFR) (Araki et al., 2012)	Locally advanced or metastatic non-small cell lung cancer (Sanford and Scott, 2009)	Hypoglycaemic (Brooks, 2012)
Sunitinib	Inhibits the VEGFR, platelet-derived growth factor receptor, and c-Kit (Le Tourneau et al., 2007)	Treatment of renal cell carcinoma and imatinib-resistant gastrointestinal stromal tumour (Sunitinib, 2007)	Hypoglycaemic (Oh et al., 2012)
Dasatinib	Inhibits BCR-ABL, SRC family, and PDGFRβ (Lindauer and Hochhaus, 2018)	Treatment of Philadelphia chromosome positive acute lymphoblastic leukaemia or chronic myeloid leukaemia (Kantarjian et al., 2006)	Hypoglycaemic (Agostino et al., 2011)

### 3.4 Tyrosine kinase inhibitors and their glycaemic effects

Studies have shown that some small molecule tyrosine kinase inhibitors such as imatinib, erlotinib, and sunitinib exhibit antihyperglycemic effects, the mechanisms of which are not yet fully understood (Prada and Saad, 2013). However, certain tyrosine kinase inhibitors like nilotinib have been observed to cause hyperglycaemia (Breccia et al., 2007). Understanding how these drugs cause antihyperglycemic effects could provide further insight on glucose handling of pharmacological targets and shed light on whether tyrosine inhibitors could be useful as pharmacophores to develop novel agents towards the management of DM. Small molecule tyrosine kinase inhibitors have been shown to reverse or even prevent type 1 and type 2 DM by reducing insulin resistance and improving β cell dysfunction, thus reversing hyperglycaemia (Fountas et al., 2015). Literature indicates that tyrosine kinase inhibitors can cause hypoglycaemia in both type 1 and type 2 DM (Hadova et al., 2021). Improvement in HbA1c and glycaemia has been noted in several cases, leading to either termination or reduction of insulin therapy when patients are being treated with imatinib and sunitinib (Buffier et al., 2018). A retrospective study investigated the blood glucose concentration in 17 diabetic and 61 non-diabetic patients being treated with imatinib, sunitinib, dasatinib, and sorafenib. A mean decline in blood glucose concentration was observed with the use of all four drugs; imatinib had a mean decline of blood glucose concentration of 9 mg/dL, sunitinib 14 mg/dL, dasatinib 53 mg/dL, and sorafenib 12 mg/dL. The decrease in blood glucose concentration observed in this retrospective study was statistically significant and it was noted that 47% of patients with diabetes were able to successfully discontinue their diabetic treatment including insulin therapy (Agostino et al., 2011).

This literature is envisaged to provide and consolidate evidence of glycaemic aberrations associated with the small molecule tyrosine kinase inhibitors. We envisage that corroborating retrospective evidence together *in vivo* and *in vitro* observation may assist in shedding deeper mechanistic insight on how small molecule tyrosine kinase inhibitors modulate glucose handling. As mentioned in the introduction, some small kinase inhibitors exhibit hyperglycaemic effects, hence understanding and differentiating the mechanisms of actions of small molecule tyrosine kinase inhibitors is crucial. This may pave the way for the repurposing of these drugs in diabetes management. We envisage discussion in the review will provide a motive for further research and development to explore the worthiness of repurposing small molecule tyrosine kinase inhibitors for the management of DM. In the next section, we present a discussion of the small molecule tyrosine kinase inhibitors outlining the indications and highlighting the known glycaemic effects to date, of each one. Table 1 provides a summary of some small molecule tyrosine kinase inhibitors investigated for their antidiabetic activity.

#### 3.4.1 Imatinib

Imatinib was approved in 2001 by the FDA after successful clinical trials for the treatment of all phases of Philadelphia chromosome positive (Ph^+^) chronic myeloid leukaemia. It was then approved for the treatment of gastrointestinal stromal tumours (GIST) in 2002 (Druker et al., 2001; Ayala-Aguilera et al., 2022). Imatinib is a selective and potent inhibitor of the protein tyrosine kinase BCR-ABL, KIT and platelet derived growth factor receptors (PDGFR-α and PDGFR-β) (Peng et al., 2005). The protein tyrosine kinase BCR-ABL is a ligand independent (constitutively) activated tyrosine kinase that causes myeloid leukaemia (CML) (Druker et al., 2001). Imatinib is a type II tyrosine kinase inhibitor and a phenylamino-pyrimidine derivative (Roskoski, 2016). After binding to the ATP binding site of the ABL domain of the BCR-ABL tyrosine kinase protein, imatinib traps the chimeric protein in an inactive conformation thus inhibiting it with nanomolar potency (Roskoski, 2003). The BCR-ABL is constitutively activated and is only present in the leukemic clone, hence imatinib’s selectivity. Imatinib inhibition of KIT and tyrosine kinase PDGFR is the basis of its selective treatment in GIST. This selectivity results in reduced side effects. Inhibition of KIT may lead to myelosuppression, dermatologic toxicity, while ABL inhibition may lead to rare cardiac events and inhibition of PDGFR may lead to altered bone metabolism and fluid retentions (Giles et al., 2009).

BCR-ABL positive cells express high affinity for the GLUT-1 glucose transporter and increased glucose uptake. This supports the theory that the control of glucose substrate flux is a vital mechanism of the antiproliferative action demonstrated by imatinib (Breccia and Alimena, 2009). The potential therapeutic benefit of imatinib in GIST cells is via decreased glucose uptake (Stroobants et al., 2003; Verweij et al., 2004). Treatment of BCR-ABL positive cells with imatinib resulted in approximately 90% of the cell surface transporter GLUT-1 being internalized (Barnes et al., 2005). Furthermore, imatinib treatment has been found to significantly reduce glucose uptake by reduced plasma membrane-bound GLUT-4 in GIST cells (Tarn et al., 2006). Boren et al. (Boren et al., 2001) have shown that imatinib treatment might reduce *de novo* fatty acid and nucleic acid synthesis through restriction of glucose-6phosphate 1- dehydrogenase activities and hexokinase in myeloid tumour cells. In a study by Gottschalk et al. (Gottschalk et al., 2004), human BCR-ABL positive cell lines treated with imatinib 0.1–1.0 μmol/L demonstrated a decrease in glucose uptake from the media via the suppression of glycolytic cell activity and increased mitochondrial Krebs cycle activity. However, we should note that these observations are reported in cancerous cells, which could suggest that a poor-glucose uptake as a cytotoxic tool to kill cancerous cells.

In a clinical observation of seven diabetic chronic myeloid leukaemia patients being treated with imatinib, six patients showed improvement in fasting blood glucose concentration which led to a reduction of their insulin dosage or oral antidiabetic drugs (Breccia et al., 2004). Imatinib has been shown to increase insulin sensitivity, as studies have shown that imatinib treatment led to stable insulin plasma levels and progressive reduction of HbA1c fraction (Breccia et al., 2005). The exact molecular mechanisms in which imatinib confers its antidiabetic properties is not clearly understood and this is a strong motive of this paper. So far, the proposed hypothesis of how imatinib improves glycaemia include preservation of beta cells viability and mass. Imatinib is thought to inhibit beta cell apoptosis through increased activation of NF-KB, an antiapoptotic factor and via decreased activation of proapoptotic MAPK JNK. Insulin sensitivity is improved via inhibition of tumour necrosis factor-α production (Wolf et al., 2005). This is believed to occur via imatinib’s inhibition of the non-receptor tyrosine kinase c-Abl (Breccia et al., 2004). Studies show that moderate dose of imatinib efficiently neutralize high-fat induced peripheral insulin resistance (Hägerkvist et al., 2007). Increased insulin sensitivity may also be due to imatinib’s slimming effect (Breccia et al., 2005; Hägerkvist et al., 2007). Imatinib inhibits phosphorylation of several kinases such as extracellular regulated kinase 1 and 2 (ERK1 and ERK2), AKT and PDGFR. This inhibition of phosphorylation leads to better signaling and functioning of effectors with improvements in insulin sensitivity (Veneri et al., 2005).

#### 3.4.2 Axitinib

Axitinib is a small molecule tyrosine kinase inhibitors derived from indazole. It is an oral multitargeted potent tyrosine kinase receptor inhibitor. Axitinib selectively inhibits VEGFR-1, -2 and -3 at sub nanomolar concentrations *in vitro* (Escudier and Gore, 2011). Axitinib was approved by the FDA for the treatment of advanced renal cell carcinoma in 2012 (Ayala-Aguilera et al., 2022). It is a second generation type IIA receptor inhibitor of VEGFR with sub nanomolar potency (Roskoski, 2016). The VEGFR is a component of the VEGF/VEGFR signaling pathways which play a role in vascular development during embryogenesis and angiogenesis during adulthood. These pathways are also implicated in growth metastasis and survival of tumours (Chen et al., 2013). Axitinib binds to intracellular tyrosine kinase domain of VEGFR and inhibits autophosphorylation of VEGFRs thus blocking downstream signaling pathways, cell survival and tube formation at picomolar concentration (Hu-Lowe et al., 2008). Axitinib is more selective against VEGFR tyrosine kinase than the tyrosine kinase inhibitors such as soraferinib, sunitinib and pazopib, as they are multitargeting (Bhargava and Robinson, 2011). Tyrrell and Pwint (Tyrrell and Pwint, 2014) observed a decrease in blood glucose concentrations and HbA1c level in a patient receiving axitinib, 5 mg twice daily. *In vitro* studies by Hudson et al. (2014) showed that axitinib increases [C-14] deoxy glucose uptake and translocation of GLUT-1 transporters from cytosolic pools to the cell surface membrane in pancreatic adenocarcinoma (PDAC) cells.

#### 3.4.3 Sunitinib

Sunitinib is a potent orally bioavailable ATP-competitive type I1/2B inhibitor. It is a multitargeted receptor tyrosine kinase inhibitor of PDGFR-α and β, FMS-like tyrosine kinase 3, VEGFR-1, -2 and 3, colony stimulating factor 1 receptor and glial cell-line derived neurotrophic factor receptor (Blay, 2010). These receptors are associated with tumour growth and angiogenesis. Sunitinib is used in the treatment of renal cell carcinoma and gastrointestinal stromal tumours (Gan et al., 2009). The development of sunitinib was brought by the development of resistance to imatinib in gastrointestinal stromal tumour potent caused by mutation of KIT (Atkins et al., 2006). Sunitinib forms polar bonds with Asp86, Glu81 and Lue83 in the CDK2 hinge region (Roskoski, 2016).

In a study that was done at Seoul National University Bundang Hospital in the Republic of Korea, 10 out of 48 diabetic patients with metastatic renal cell carcinoma, were reported to have a significant decrease in blood glucose levels after treatment with sunitinib dose for 4 weeks. However, their blood glucose concentrations reverted to normal 2 weeks after treatment cessation. Sunitinib doses were not disclosed by the authors, however since no special note was highlighted, we propose that the doses were within the global recommendations of 50 mg daily for an adult patient. One of the 10 patients was able to discontinue their diabetic medication and 3 patients had dose reductions of their oral antidiabetics during the 4-week sunitinib treatment. Conversely, 38 non-diabetic patients showed no significant changes in blood glucose levels during the 4 weeks of sunitinib treatment (Oh et al., 2012). In a retrospective review, all 19 types 2 diabetes mellitus patients showed a reduction in blood glucose concentrations after 4 weeks sunitinib treatment. Some studies reported discontinuation of diabetic medications as a results of sunitinib treatments, whilst others reported patients having normalized blood glucose (Billemont et al., 2008).

The exact blood glucose lowering mechanism of tyrosine kinase inhibitors is unknown. It has been suggested that tyrosine kinase inhibitors’ effect on c-KIT tyrosine kinase is in part responsible for the alteration in blood glucose levels. C-KIT is associated with β-cell survival (Rachdi et al., 2001). Sunitinib is believed to have a direct effect on β-cells resulting in glucose-induced insulin secretion which leads to lowered glucose in patients. Sunitinib stimulates insulin secretion in a concentration and dose dependent manner (Lutz et al., 2017). Billemont et al. (2008) suggested that sunitinib affects insulin resistance by interfering with insulin-like growth factor-1 pathway. Louvet et al. (2008) using data from mouse models suggested that inhibition of PDGF downstream-mediated inflammatory response improves pancreatic β-cell survival and insulin resistance.

#### 3.4.4 Nilotinib

Nilotinib is an orally administered type IIA BCR-ABL inhibitor, second generation tyrosine kinase inhibitor developed by Novartis for treatment against imatinib-resistant mutants of BCR-ABL. Nilotinib binds to the inactive DFG-out conformation of the BCR-ABL (Weisberg et al., 2006). Nilotinib is a high-affinity aminopyrimidine-based ATP competitive inhibitor that reduces cell proliferation and viability through selective inhibition of BCR-ABL autophosphorylation (Weisberg et al., 2005). To maximize efficacy, nilotinib was designed to bind more tightly to the BCR-ABL than imatinib, by incorporating a N-methypiperamine moiety while keeping the amide pharmacophore to preserve the H-bond interaction to Glu286 and Asp381. Hence nilotinib is able to maintain binding to the inactive conformation of the ABL kinase domain (Golemovic et al., 2005). Out of the 33 imatinib-resistant BCR-ABL mutants, nilotinib is active against 32 except for the T315I mutant. Nilotinib has more lipophilic interactions than imatinib; it has 4 hydrogen-bond interactions with the ABL kinase domain, which results in nilotinib expressing higher affinity for binding than imatinib (Manley et al., 2005).

Nilotinib and imatinib are similar in structure but reports have shown them to have opposite effects on glucose metabolism (Breccia et al., 2007). Iurlo et al. (2015) observed that patients on nilotinib had significantly higher levels of insulin, C-peptide, fasting plasma glucose, insulin resistance and LDL cholesterol compared to patients that were being treated with imatinib and dasatinib. It was however concluded that nilotinib treatment does not induce diabetes mellitus or impair fasting glucose to a significantly higher extent than imatinib and dasatinib, but nilotinib causes a worse glucometabolic profile. Hence it has been advised that patients on nilotinib require dose monitoring of glucose and lipid metabolism. A large phase III trial comparing the efficacy of nilotinib and imatinib showed that 53% of the patients being treated with nilotinib 400 mg b.i.d were observed to have hyperglycaemia, 50% of patients treated with 300 mg b.i.d and only 30% of patients treated with imatinib 400 mg/day were observed to have hyperglycaemia (Larson et al., 2014). Nine out of thirteen chronic myeloid leukaemia patients being treated with nilotinib showed increased fasting glucose levels as compared to baseline pre-nilotinib therapy (Breccia et al., 2007). In a study by Philipp le Courte an increase in grade ¾ hyperglycaemia was observed during nilotinib therapy (Larson et al., 2014). Fasting 1-h and 2-h plasma glucose concentrations increased after 3 months of nilotinib treatment (Racil et al., 2013). Based of fasting glucose (8.1 and 7.3 mmol/L), 2 patients fulfilled the criteria of diabetes mellitus while on nilotinib therapy and 2 patients had impaired glucose tolerance. It is worth noting that most studies have concluded that the hyperglycaemia caused by nilotinib is clinically insignificant as it is usually mild, transient and manageable (Saglio et al., 2010). However, understanding the mechanism by which the hyperglycaemia is brought about is crucial, especially for patients with both chronic myeloid leukaemia and diabetes mellitus.

The mechanism in which nilotinib impairs glucose metabolism is not clearly known. In one case of pre-existing severe type 2 diabetes mellitus, nilotinib was shown to directly affect glucose metabolism via impairment of endogenous insulin secretion. When nilotinib was discontinued the impairment to endogenous insulin secretion was reserved (Ito et al., 2013). Insulin resistance which was calculated by the mathematical model HOMA2-IR increased significantly during nilotinib therapy (Racil et al., 2013). Insulin sensitivity index HOMA2-%S significantly decreased as well as ISI0,120. HOMA2-%S and HOMA2IR represent hepatic insulin resistance while ISI0,120 represents peripheral insulin resistance. The cABL domain is associated with the insulin signaling pathway. It has been hypothesized that by blocking the c-ABL, tyrosine kinase therapy affects the insulin receptor pathway (Genua et al., 2009). Plasma concentration of adiponectin has been observed to have decreased during nilotinib therapy. Adiponectin exerts a potent insulin-sensitizing effect (Shehzad et al., 2012; Racil et al., 2013).

#### 3.4.5 Gefitinib

Gefitinib is a 4-anilinoquinazoline derivative, a potent and selective ATP competitive inhibitor of EGFR and HER-2 kinases (Rahman et al., 2014). Gefitinib binds to the ATP binding pockets of EGFR’s active form, hence it is classified as a Type-I kinase inhibitor. Gefitinib forms H-bonds with Met793 in the hinge region (Ayala-Aguilera et al., 2022). Mutations in the extracellular segment of the EGFR such as deletion have been shown to result in constitutive growth factor independent EGFR activation leading to the overexpression of the EGFR, which causes the progression of numerous malignancies (Ekstrand et al., 1992). A common mutation of the EGFR is the L858R which results from exon 19 substitution of leucine with arginine, and this accounts for 40% of mutations in non-small cell lung cancer. The other mutation is exon 19 deletion. These mutations cluster around the active site cleft of the tyrosine kinase domain of the EGFR (Chan et al., 2006). Studies of direct binding measurements have shown that gefitinib binds 20-fold more tightly to the L858R mutant form of the EGFR as compared to the wild type receptors. Cells with mutant EGFR tend to be more sensitive to tyrosine kinase inhibitors than cells that express the wild type kinase (Yun et al., 2007). Gefitinib is considered to be relatively selective, however it demonstrates moderate activity in other receptor tyrosine kinase resulting in common adverse events such as diarrhoea, rash, nausea and dry skin (Culy and Faulds, 2002). In 2004 gefitinib became the first epidermal growth factor receptor tyrosine kinase inhibitor for molecular targeted chemotherapy of unresectable non-small cell lung cancer in Japan. The antineoplastic effect of gefitinib comes from inhibiting the activation of ErbB-1 (EGFR) (Araki et al., 2012). Clinically gefitinib is used for treatment of chemo resistant non-small cell lung cancer (Rahman et al., 2014).

Gefitinib has been shown to have a dramatic effect on a limited number of patients. Studies have suggested that it is ineffective in 70%–80% of patients with non-small cell lung cancer (Fukuoka et al., 2023). EGFR inhibitors, erlotinib and gefitinib seem to have secondary pharmacological mechanisms of action, as they appear to be inhibitors of Tumour Necrosis Factor Alpha (TNF-α). They may prevent progression of type 1 diabetes mellitus as they appear to be effective in the treatment of related TNF-α mediated inflammatory diseases. This is achieved through the inhibition of the TNF-α (Brooks, 2012). Inflammation associated with TNF-α has been linked to the development of insulin resistance (Borst, 2004). Gefitinib may be of value in the treatment of type I diabetes mellitus by inhibition of TNF-α, leading to reduction in inflammation, decreased insulin resistance and slowed progression of Beta-cell destruction (Brooks, 2012).

In a rat model study, gefitinib improved insulin sensitivity in rats with type 2 diabetes. This is hypothesised to occur through the increase in PI3K/AKT pathway of insulin signaling (Sun et al., 2009). Gefitinib has inhibitory action on receptor-interacting protein kinase 2 (RIPK2). RIPK2 propagate immune responses that are associated with dysglycemia during obesity and insulin resistance (Tigno-Aranjuez et al., 2014; Denou et al., 2015). In a mice model study, gefitinib improved glucose control independently of RIPK2. In the absence of RIPK2 gefitinib improved insulin sensitivity and lowered insulin secretion (Duggan et al., 2020). One study showed that there was a significant decrease in fluorodeoxyglucose (FDG) uptake in gefitinib sensitive cell lines (Su et al., 2006). FDG uptake is a marker for tissue uptake of glucose (Ashraf and Goyal, 2023). In the same study immunoblots showed the translocation of GLUT3 from the plasma to the cytosol (Su et al., 2006).

#### 3.4.6 Dasatinib

Dasatinib is a type 1 inhibitor of ABL tyrosine kinase and a type I1/2A inhibitor of LYN. It is an orally available ABL inhibitor which is different to imatinib such that it binds to both the active and inactive conformations of the ABL kinase domain (Talpaz et al., 2006). In 2006, the FDA granted accelerated approval for the treatment of chronic myeloid leukaemia and Ph+ acute lymphoblastic leukaemia (Ph+ ALL) with dasatinib in patients that had demonstrated resistance or intolerance to prior therapy with imatinib (Kantarjian et al., 2006; Jabbour and Lipton, 2013). Resistance in the large proportion of patients on imatinib therapy is a result of point mutations of the BCR-ABL gene and activation of alternative signaling pathways the likes of SRC family kinase (Kantarjian et al., 2006). Dasatinib is a potent ATP competitive inhibitor, it has less stringent binding requirements as compared to imatinib; hence it shows inhibition activity against many imatinib resistant BCR-ABL mutations. Dasatinib is active against 14 out of 15 imatinib resistant BCR-ABL mutants (Shah et al., 2004). The one mutant not inhibited by dasatinib is the T315I mutant, which is a single mutation deep within the ATP-biding pockets of the ABL tyrosine kinase (Weisberg et al., 2005). Dasatinib is a multikinase inhibitor that shows activity against PDGFR- β, KIT, and SRC family kinase, and because of this range of inhibition, dasatinib has many side effects including haemorrhage, teratogenicity, fluid retention and myelosuppression (Steegmann et al., 2012).

In a retrospective study on blood glucose concentrations in eight diabetic and non-diabetic patients treated with dasatinib, a mean decrease in blood glucose of 53 mg/dL was observed. Some patients on dasatinib treatment were able to discontinue their antidiabetic medication (Agostino et al., 2011). A case report of a 57-year-old diabetic chronic myeloid leukaemia patient had their insulin requirements declined upon the introduction of dasatinib. Within 2 weeks of dasatinib treatment, insulin treatment was discontinued. The patient’s fasting C-peptide immunoreactivity increased two-fold, suggesting that dasatinib allowed for the recovery of insulin production (Ono et al., 2012). In another case report, a 65-year-old woman with type 2 diabetes mellitus, had her glycaemic index improved to <6.0% upon dasatinib treatment initiation. Quantitative insulin sensitivity check index rapidly improved followed by an improvement in insulin sensitivity. This case report demonstrated that dasatinib might have negative as well as positive effects on glucose metabolism (Iizuka et al., 2016). In a retrospective study, patients that were treated with dasatinib had a mean decrease in HbA1c of 0.80 absolute point and these patients required 8.2 less total daily insulin units on average. This led to a conclusion that dasatinib demonstrates antidiabetic effects and may be considered for the use as a novel diabetic therapy. The mechanism by which dasatinib instigates these antidiabetic effects have not been stated and remains unknown (Salaami et al., 2021).

Evidence suggests that tyrosine kinase play a role in regulating insulin secretion. Various mechanisms underpin the involvement of SRC kinase, including their potential regulation through myristylation, dynamic palmitoylation, and acylation. All which contribute to the inhibition of insulin secretion (Breccia et al., 2008). SRC kinase pathways are integral in the extracellular calcium-dependent secretion of pancreatic enzymes and insulin (Cheng et al., 2007). Dasatinib’s inhibition of SRC kinase pathways leads to the reduction of fasting glucose observed in the studies mentioned.

**FIGURE 3:**
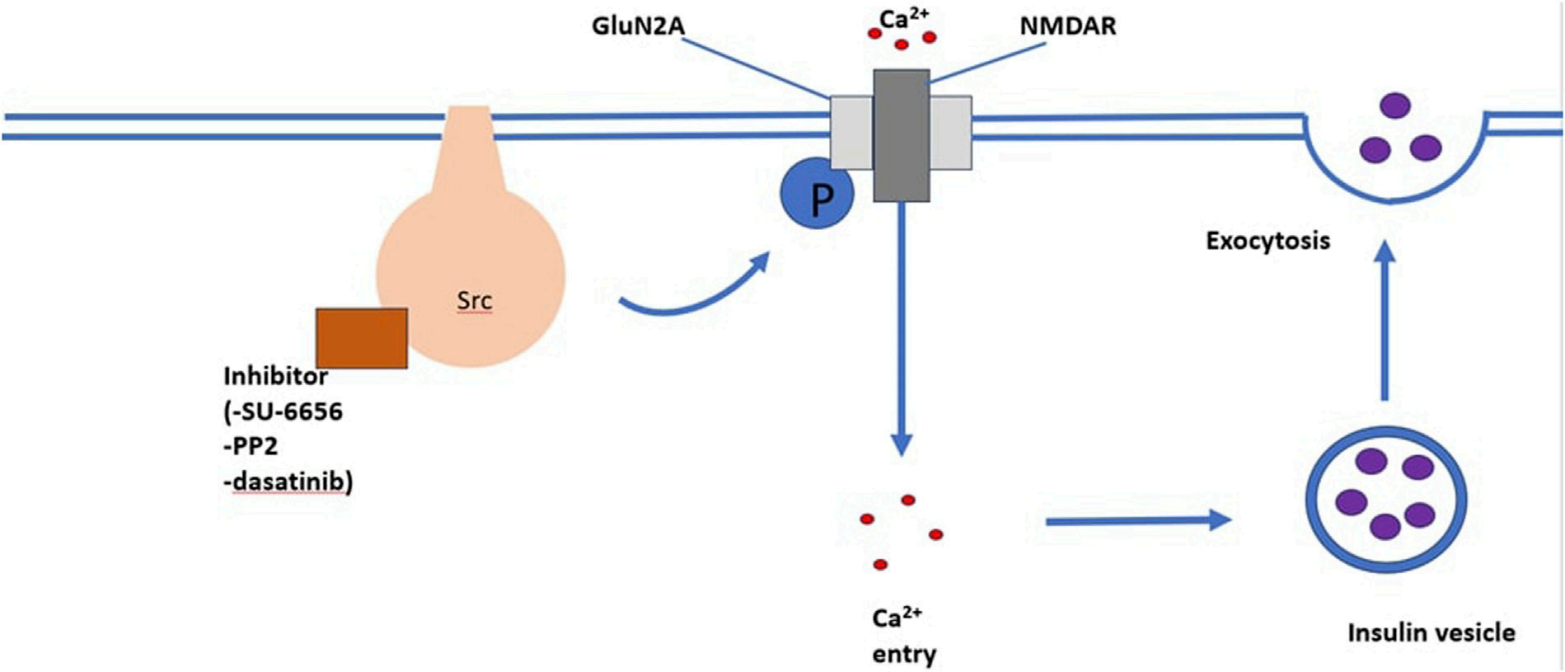
An illustration of the mechanism through which the SRC-family kinase pathway enhances insulin secretion. The binding of inhibitors such as SU-6656, PP2, and dasatinib enhances Ca2+ induced insulin secretion.

SRC-family kinase are proto-oncogenes that play a role in cell processes, including cell differentiation, cell growth, cell morphology, mortality and survival (Parsons and Parsons, 2004). Insulin secretion and neurotransmitter release both occur via exocytosis (Cheng et al., 2003; Baldwin et al., 2006). Exocytosis is an ubiquitously occurring mechanism responsible for cell to cell communication (SIM et al., 2003). It has been suggested that SRC-family tyrosine kinase inhibit neurotransmitter release and it is believed that insulin secretion and neurotransmitter release have similar regulatory mechanisms. Cheng et al. (2007) observed that SU-6656 and PP2, structurally different SRC-family tyrosine kinase inhibitors, enhanced Ca^2+^-induced insulin secretion in INS-1 cells and rat pancreatic islets in both a time and dose dependent manner. This study illustrates that one or more SRC-family tyrosine kinase exert a tonic inhibitory role on Ca^2+^-dependent insulin secretion. The role of SRC-family tyrosine kinase in Ca^2+^-induced insulin secretion is illustrated in Figure 3.

SRC-family tyrosine kinase are non-receptor tyrosine kinase. They are expressed in high levels on secondary vesicles and plasma membranes of cells that are specialised for exocytosis. Cells which fall under this category include neuronal and endocrine cells (Ely et al., 1994). Evidence from studies suggests that protein tyrosine kinase are associated with the regulation of insulin secretion (Sorenson et al., 1994). Small molecule tyrosine kinase inhibitors have been observed to induce increases in insulin secretion in INS-1 cells (Verspohl et al., 1995). On the other hand, some studies have reported decreased insulin secretion due to small molecule tyrosine kinase inhibitors in adult rat islets (Persaud et al., 1999). These varying observations highlight that different protein tyrosine kinases have different or opposing outcomes on insulin secretion. The exact function of protein tyrosine kinase in insulin secretion is unknown.

## 4 Authors perspective and recommendations

The development of small molecule tyrosine kinase inhibitors has revolutionized the cancer treatment landscape. Small molecule kinase inhibitors have been developed for targeted cancer therapy, hence the increase in their popularity and use. Despite their great potential, there aremajor challenges pertaining to toxicity and resistance. Small molecule tyrosine kinase inhibitors have been linked to altered glucose metabolism in numerous studies, with some small molecule tyrosine kinase inhibitors inducing hyperglycaemia including nilotinib, ceritinib and rociletinib. The hyperglycaemia induced have been noted as one of their dose-limiting side effects which has been attributed to impaired pancreatic β-cell insulin secretion or development of insulin resistance and inhibition of the insulin receptor. However, studies have shown diabetic cancer patients on small molecule tyrosine kinase inhibitors demonstrate improved glycaemic control and require a reduction in their daily insulin requirements or diabetic medication in both type 1 and type 2 diabetes. Examples of such kinase inhibitors include sunitinib, imatinib, and erlotinib. The improved blood glucose concentration is achieved potentially in part through preserving functional βcell mass and increased insulin sensitivity or insulin secretion.

The exact mechanism by which small molecule tyrosine kinase inhibitors elicit an increase or a decrease in patients largely remains elusive and unknown. Given their role and increase in use, this is a cause for concern, particularly in hospitalized comorbid patients, as slight alterations in blood glucose may result in complications or mortality. Research into the mechanism needs to be prioritized, with the aim to shed light and assist prescribers with the administration of treatment which will offer the patients the best possible prognosis.

Some authors have highlighted small molecule tyrosine kinase inhibitors potential as novel therapies for diabetes (Prada and Saad, 2013; Fountas et al., 2015). Considering the increasing incidence and prevalence of diabetes, the novel therapies school of thought is a well-grounded approach. However, before we explore their potential use as antidiabetics, there is a necessity to fully elucidate their glycaemic altering mechanism at both cellular and molecular level. There is also a necessity to establish discrepancies observed amongst small molecule tyrosine kinase inhibitors in the context of glycaemic control. Perhaps the answer lays in the chemical structural differences. This includes uncovering whether the alteration is dependent on the type of the small molecule tyrosine kinase inhibitors. In the short term, this will be a valuable tool for physicians, prescribers, and other healthcare professionals. For example, diabetic patients with cancer being treated by small molecule tyrosine kinase inhibitors may need to be assigned to those that are known to improve blood glucose concentration. This may have potential benefits including a reduction in polypharmacy which can prove to have precipitate adverse effects. Our line of thought can be backed by studies which indicated that some patients on small molecule tyrosine kinase inhibitors required dose reductions in their diabetic medication with some case patients discontinuing diabetes treatment while under a small molecule kinase inhibitor therapy. With the opposite being true as well, patients at risk of hyperglycaemia complications including neuropathy or kidney damage may need to be kept away from small molecule tyrosine kinase inhibitors known to induce hyperglycaemia.

These strategies are only possible if the glycaemic-altering mechanisms are known, hence further research into the inner workings of small molecule tyrosine kinase inhibitors is a necessity for the healthcare system. Uncovering how glucose metabolism is altered by small molecule tyrosine kinase inhibitors will provide more insight into glycaemic control in cancer patients. This will not only open the possibility of potential alternative methods to manage glycaemic dysregulation, but it may shed light on what is currently known about the pathophysiology of diabetes and cancer. This is of paramount importance given the global demography of an aging population. It is well documented that the incidence of morbidities such as cancer and diabetes is higher in the geriatric population as compared to the others.

The long-term effects of the altered glucose metabolism by small molecule tyrosine kinase inhibitors are poorly documented. There is a necessity to elucidate whether the cases of glycaemic dysregulation in patients are isolated events that are due to particular conditions. For instance, certain morbidities render patients more susceptible to phenomena which individuals where these morbidities are absent are not as susceptible. This calls for large scale follow up studies and not just limit our understanding to retrospective case studies. This would help uncover if the effects elicited by the small molecule tyrosine kinase inhibitors are limited to a particular demography. In the event that the effects can be expected in the general population, the relevant healthcare professionals, especially oncologists need to be informed accordingly for the safe and optimum use of these compounds.

Another vital area of research is to uncover the factors that may sensitize a patient to glycaemic dysregulation observed in small molecule tyrosine kinase inhibitors. Highlighting these factors before commencing therapy has the potential to improve patient health outcomes and possibly reduce the number of hospital days.

Existing evidence suggests that small molecule tyrosine kinase inhibitors have a lot of potential as novel therapy for DM. This is not the case with all but small molecule tyrosine kinase inhibitors by way of example imatinib. The chronic nature of DM costs and side effects are major concerns when proposing the use of small molecule tyrosine kinase inhibitors in DM treatment. The estimated cost of small molecule tyrosine kinase inhibitors for chronic myeloid leukaemia ranges between $3 632 and $8 429 USD (Talon et al., 2021). While according to drugs.com the price of a pack of 30 metformin 500 mg oral tablets is approximately $13,15 (Drugs.com, 2024a). According to drugs.com 30 tablets of 100 mg oral imatinib tablets and 28 oral tablets of 12,5 mg sunitinib have an approximate cost of $219 and $5 198–$5 492 respectively (Drugs.com, 2024b). For chronic use in the management of DM the cost-benefit ratio is not in favour of small molecule kinase inhibitors when compared to current antidiabetic agents which already have healthcare budgets across the globe stretched. The cost of small molecule tyrosine kinase inhibitors is a major obstacle which would limit their use in DM. Another concern to highlight is that the current price of small molecule tyrosine kinase inhibitors is a direct contributing factor of non-adherence mortality from certain cancers.

It has been reported that nearly all patients experience treatment related adverse events while on small molecule kinase inhibitor treatment. One study stated that up to 70% of the patients undergoing small molecule tyrosine kinase inhibitors treatment experienced adverse events. Caldemeyer et al. (2016) reported that 60% of the patients on imatinib treatment experienced oedema, 50% nausea, 49% muscle cramps, and 47% musculoskeletal pain. However overall small molecule tyrosine kinase inhibitors appear to be well tolerated with patients experiencing mild or moderate adverse events. Some common adverse events include skin toxicity, diarrhoea, and fatigue (Hartmann et al., 2009; Caldemeyer et al., 2016; Płużański and Piórek, 2016). Adverse events led to discontinuation of imatinib in only 4% of patients in some studies (Druker et al., 2006). Small molecule tyrosine kinase inhibitors share the same mechanism of action of competitive inhibition of ATP at the catalytic site of tyrosine kinase. However, they differ in the specific tyrosine kinase they target, hence they elicit the varied pharmacological responses and side effects. This difference opens the possibility for targeted therapy and minimal side effects with regards to their use in DM management.

Indeed, understanding the mechanism of action at the molecular level is a starting point, which should then be followed by elucidating the structure activity relationships. Deeper understanding of structure activity relationships will allow these compounds to be modified accordingly. As mentioned earlier, the mechanism of action of small molecule tyrosine kinase inhibitors is largely the same (ATP competitive inhibition), while the differences are conferred by the spectrum of targeted tyrosine kinases. Elucidating why certain small molecule tyrosine kinase inhibitors have higher affinity for specific tyrosine kinase as compared to others is key. Small molecule tyrosine kinase inhibitors effect on glucose metabolism is strongly linked to the tyrosine kinase they interact with. Structural modification of small molecule tyrosine kinase inhibitors paves the way for specificity in terms of the tyrosine kinase they will interact strongly with. This in turn may be utilized to optimise their role in glucose metabolism.

## 5 Conclusion

In conclusion, the development of small molecule tyrosine kinase inhibitors has undoubtedly transformed the landscape of cancer treatment, offering new hope for targeted therapy. Small molecule tyrosine kinase inhibitors have however been associated with glycaemic dysregulation in patients. With some small molecule tyrosine kinase inhibitors improving glycaemic regulation in diabetic patients while others induce clinically insignificant hyperglycaemia. The focus of this paper aimed to understand small molecule tyrosine kinase inhibitors on glycaemic control and their mechanisms with the goal of improving patient health outcomes and prognosis. This is vital given the incidence and prevalence of diabetes and cancer. As the global population ages and the incidence of cancer and diabetes continues to rise, understanding the complex interplay between kinase inhibitors and glucose metabolism is not just a medical imperative but a fundamental step toward advancing our knowledge of the pathophysiology of these diseases. Research in this field holds the promise of improving patient care, optimizing treatment strategies, and enhancing the overall quality of healthcare for a growing demographic in need of effective therapeutic solutions. Furthermore, considering the prevalence of diabetes, it would be worthwhile for researchers to explore repurposing strategies of small molecule tyrosine kinase inhibitors towards diabetes mellitus.
